# Association between grit and depressive symptoms among medical students, moderated by academic performance

**DOI:** 10.1080/10872981.2024.2373523

**Published:** 2024-07-01

**Authors:** Mitsuyuki Numasawa, Nobutoshi Nawa, Kumiko Yamaguchi, Keiichi Akita, Masanaga Yamawaki

**Affiliations:** a Institute of Education, Tokyo Medical and Dental University, Tokyo, Japan; b Department of Medical Education Research and Development, Tokyo Medical and Dental University, Tokyo, Japan; c Department of Global Health Promotion, Tokyo Medical and Dental University, Tokyo, Japan; d Department of Clinical Anatomy, Tokyo Medical and Dental University, Tokyo, Japan

**Keywords:** Medical students, depressive symptoms, depression, mental health, grit, perseverance of effort, consistency of interest, academic performance, academic achievement

## Abstract

Depression amongst medical students is a crucial matter. Grit, which is a potentially modifiable psychological factor, has been inversely linked to depressive symptoms. However, it remains unclear how grit is associated with depression. This study aims to examine the relationship between grit and depressive symptoms and to further investigate the potential effect modification by academic performance on the association between grit and depression among medical students. We focus on the total grit score and its subscales, namely perseverance of effort and consistency of interest. A cross-sectional study was conducted using data from second-year medical students at Tokyo Medical and Dental University in Japan from 2020 to 2023. The participants responded to questionnaire surveys comprising the Center for Epidemiologic Studies Depression Scale and the Short Grit Scale. Linear regression analysis was performed to assess the association between grit and depressive symptoms. We also tested for effect modification by first-year Grade Point Average (GPA) on the association between grit and depression. The total grit score and its subscales, perseverance of effort and consistency of interest, were all inversely associated with depressive symptoms (b =  −4.7 [95%CI − 6.7 to − 2.6], b =  −3.7 [95%CI − 5.3 to − 2.1], b =  −1.8 [95%CI − 3.5 to − 0.2], respectively). While the interaction term for the total grit score and GPA was not significant, the interaction term for perseverance of effort and GPA was significant, indicating that the association between perseverance of effort and depression was stronger among the higher-achieving students. The interaction term for consistency of interest and GPA was also significant, indicating that the association was stronger among the lower-achieving students. We reveal a novel aspect of the association between grit and depressive symptoms in light of academic performance. The findings will contribute to future research on depression amongst medical students.

## Introduction

The mental health of medical students is a crucial issue [1–4]. Depression is one of the most common mental disorders causing severe symptoms [5,6]. It can affect a person’s feelings, thoughts, daily activities, and relationships within the community [5,6]. It is also associated with suicidal risk [7,8]. About 280 million people in the world suffer from depression, and an estimated 5% of adults experience depression [5]. The prevalence of depression in university students is considerably higher than that in the general population [9]. Several studies reveal that medical students experience depression at a higher rate than both the general population and peers of a similar age [2–4]. Some studies suggest that depression prevalence is especially high in the earlier years of medical school [1,10,11]. One of those studies showed significant worsening of depressive symptoms from first year to second year [11]. The psychosocial stressors unique to university students may increase the prevalence of depression [12–15], since risk factors for depression include stress and stressful events [5,6]. Because Japanese medical students face a very full curriculum in the Japanese medical education system [16], academic burden may count as a psychosocial stressor. To produce medical doctors who can maintain the healthcare system, educators need to consider how they can help medical students avoid depression and complete their studies normally. Therefore, depression amongst medical students is a crucial issue not only as a disease to treat in a healthcare setting but also in the context of medical education.

Psychological factors contribute to the development of depression [5,6]. Thus, interventions focusing on positive psychological aspects have attracted much attention [17–20]. Several studies indicate the effectiveness of such interventions in ameliorating depressive symptoms [18–21]. In this context, grit is another noteworthy and potentially modifiable psychological factor [22,23]. The Grit Scale measures the level of perseverance and passion for long-term goals as a noncognitive trait [24–26]. Many studies report that grit is inversely associated with depressive symptoms [12,22,23,27–38]. However, few studies have focused on the association between grit and depression among medical students [31].

To establish tailored interventions focusing on grit to prevent depression among medical students, it is essential to clarify how and why grit is associated with depression. One way to elucidate this point is to identify the factors modifying the effect of grit on depression [28]. One study reported that grit mitigated the detrimental effect of emotional exhaustion on depressive symptoms [39]. Furthermore, high academic performance is achieved through overcoming stressful academic difficulties. Thus, students with higher academic performance may be able to utilize grit more effectively to cope with exhausting academic burden than students with lower academic performance. In addition, grit is positively associated with academic achievement [40–43], while depression is negatively associated with it [44–48]. Therefore, on the basis of these relationships among the three variables, it is possible that the effect of grit on depression is modified by academic performance. That is, the effect of grit on depression for high-achieving students may differ from that for low-achieving students. Clarifying how grit and its subscales are important depending on the student’s academic performance will help design tailored interventions to ameliorate depressive symptoms among medical students. Nevertheless, to our knowledge, no study has evaluated the effect modification by academic performance on the association between grit and depressive symptoms.

This study aims to examine the relationship between grit and depressive symptoms among second-year medical students in Japan. We also investigate the potential effect modification by the Grade Point Average (GPA), representing a measure of academic performance, on the association between grit and depression. Further, grit has two dimensions: perseverance of effort and consistency of interest [11,25]. Several studies suggest that the influence of these two facets of grit is different in several respects [35,40,42,49–53]. Therefore, we examine not only the total grit score but also the two subscales (i.e., perseverance of effort and consistency of interest).

## Materials and methods

### Study participants and procedure

**Figure 1.**
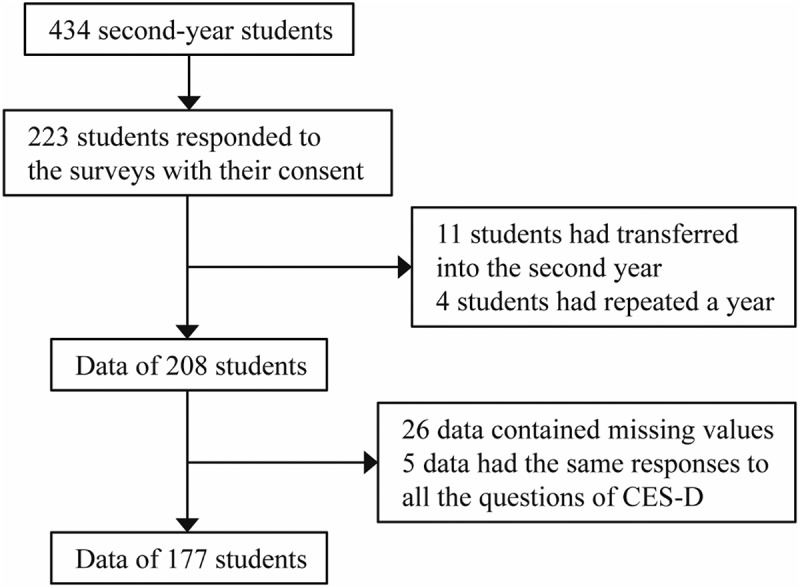
Flow chart of sampling.

We conducted a cross-sectional study using data from second-year medical students at Tokyo Medical and Dental University (TMDU) in Japan from 2020 to 2023. Questionnaire surveys were carried out in April, the first month in each academic year. The students were informed that the study was linking their responses with their corresponding academic data stored for educational purposes by the Curricular Institutional Research Division, Institute of Education at TMDU and that the students had the right to refuse to participate in the study. Of the 434 students, 223 responded to the surveys with written informed consent. The data for students who had transferred into the second year after graduating from another university and for those students who had repeated a year were excluded. The questionnaires comprised the Japanese version of the Center for Epidemiologic Studies Depression Scale (CES-D) and the Short Grit Scale (Grit-S) [25,54–56]. We excluded responses containing any missing values on the variables used for analysis, and data in which the responses to all 20 questions of CES-D were the same, noting that it includes reverse-scored items and that all the questions have an even number of choices, thus lacking a neutral point. Thus, the data of 177 participants were analyzed in the present study (Figure 1). The study was approved by the Ethics Committee at TMDU (M2019–300).

### Grit

We used the validated Japanese version of the Short Grit Scale (Grit-S) [25,56]. It comprises eight items using a five-point Likert scale ranging from 1 to 5. The possible averaged score ranges from 1 to 5, with higher scores representing higher levels of grit. Each of the two subscales, namely perseverance of effort and consistency of interest, consists of four items. The ranges of the subscales are the same as that of the total grit score. The McDonald’s omega value for the Grit-S was 0.86, indicating adequate internal consistency.

### Academic performance

We utilized the first-year Grade Point Average (GPA) to assess the students’ academic performance, which is calculated at the end of the first year of the undergraduate course.

### Covariates

Sex (male or female), age, and survey year were included as covariates, with age being a continuous variable and survey year being a categorical variable.

### Depressive symptoms

The validated Japanese version of the Center for Epidemiologic Studies Depression Scale (CES-D) was used to assess the participants’ depressive symptoms [54,55]. It is a 20-item self-rated instrument using a four-point Likert scale ranging from 0 to 3. The total score ranges from 0 to 60, with higher scores representing higher levels of depressive symptoms. The Cronbach’s alpha value for CES-D in this study was 0.88, indicating sufficient internal consistency.

### Statistical analysis

Linear regression analysis was performed to assess the association between grit and depressive symptoms, with *p* < 0.05 considered statistically significant. In addition, we tested the effect modification by the first-year GPA on the association between Grit-S and CES-D using linear regression models, with *P* for the interaction term being < 0.20 considered statistically significant. All statistical analysis was conducted using RStudio, version 2023.12.0 (Posit Software, PBC, Boston, MA, USA).

## Results

Table 1 shows the characteristics of the study participants and descriptive statistics of the variables. Of the 177 students included in the current study, 57.6% were male and 42.4% were female. The mean of CES-D was 13.2, and 33.9% of the sample had CES-D scores of ≥ 16 indicating depressive symptoms [54]. The means of the total grit score, perseverance of effort, and consistency of interest were 3.2, 3.5, and 2.8, respectively.

**Table 1.** Characteristics of study participants and descriptive statistics of variables.

Variable	*n* (%) or Mean (SD)
Year of participation	
2020	63 (35.6)
2021	50 (28.2)
2022	30 (16.9)
2023	34 (19.2)
Sex	
Male	102 (57.6)
Female	75 (42.4)
Age	19.3 (1.1)
CES-D	13.2 (8.9)
Grit-S	
Total score	3.2 (0.6)
Perseverance of effort	3.5 (0.8)
Consistency of interest	2.8 (0.8)
First-year GPA	3.5 (0.2)

Abbreviations: SD = standard deviation.

### Association between grit and depressive symptoms

Table 2 shows the association between Grit-S and CES-D by linear regression analysis. The total grit score, along with its sub-scales, perseverance of effort and consistency of interest, were all inversely associated with depressive symptoms (coefficient (b) for the total grit score = −4.7, 95%CI − 6.7 to − 2.6; b for perseverance of effort = −3.7, 95%CI − 5.3 to − 2.1; b for consistency of interest = −1.8, 95%CI − 3.5 to − 0.2). These associations remained significant after adjusting for the covariates.

**Table 2.** Association between grit and depressive symptoms.

	Crude		Adjusted^a^	
Variable	b (95%CI)	p value	b (95%CI)	p value
Total grit score	−4.7 (−6.7, −2.6)	<0.001	−4.7 (−6.7, −2.6)	<0.001
Perseverance of effort	−3.7 (−5.3, −2.1)	<0.001	−3.6 (−5.2, −2.0)	<0.001
Consistency of interest	−1.8 (−3.5, −0.2)	0.029	−1.9 (−3.5, −0.3)	0.021

Abbreviations: CI = confidence interval. ^a^Adjusted for sex, age and survey year.

### Effect modification by academic performance on the association between grit and depressive symptoms

**Figure 2.**
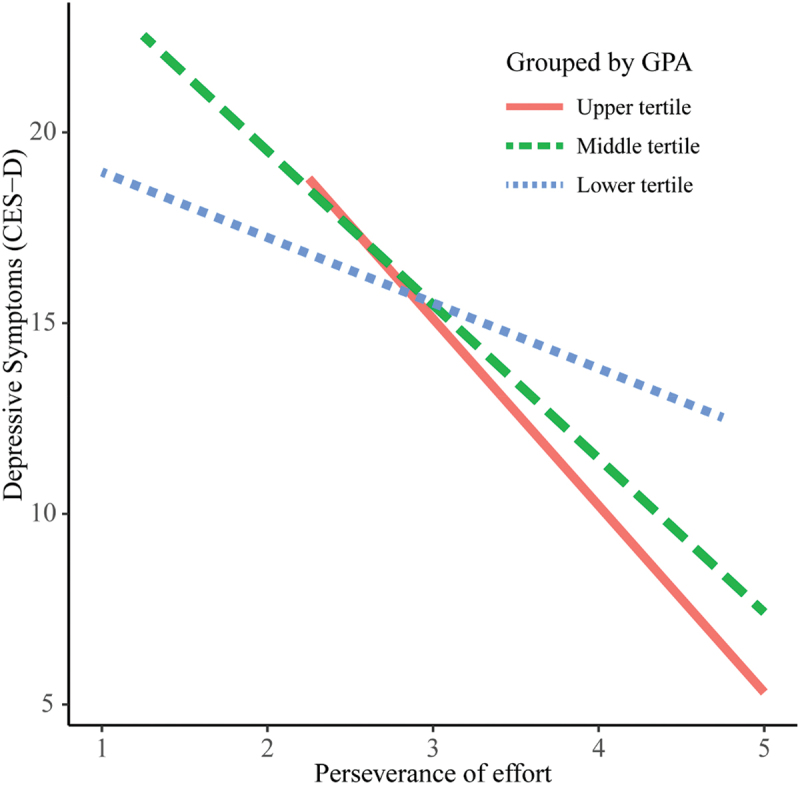
Association between perseverance of effort and depressive symptoms, grouped by academic performance.

Abbreviations: CES-D = Center for Epidemiologic Studies Depression Scale; GPA = Grade Point Average. The lines indicate the association between perseverance of effort and CES-D in the crude linear regression models, grouped by GPA: upper tertile (GPA ≥ 3.67, *n* = 59), middle tertile (3.44 ≤ GPA ≤ 3.66, *n* = 60), and lower tertile (GPA ≤ 3.43, *n* = 58). Effect modification by academic performance was significant.

**Figure 3.**
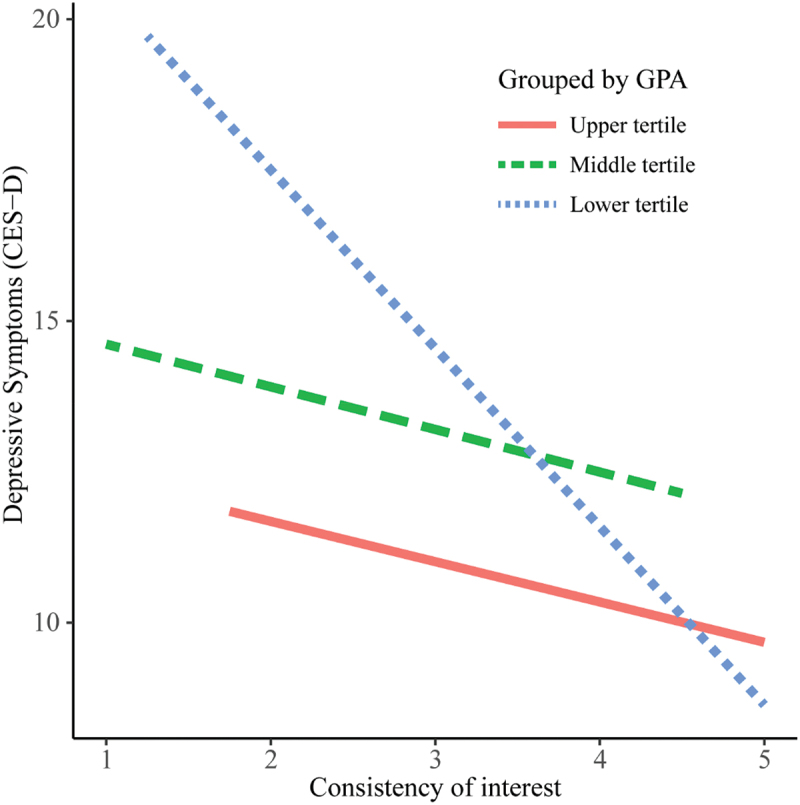
Association between consistency of interest and depressive symptoms, grouped by academic performance.

Abbreviations: CES-D = Center for Epidemiologic Studies Depression Scale; GPA = Grade Point Average. The lines indicate the association between consistency of interest and CES-D in the crude linear regression models, grouped by GPA: upper tertile (GPA ≥ 3.67, *n* = 59), middle tertile (3.44 ≤ GPA ≤ 3.66, *n* = 60), and lower tertile (GPA ≤ 3.43, *n* = 58). Effect modification by academic performance was significant.

The interaction term for the total grit score and GPA was not significant (Table 3). However, the interaction term for perseverance of effort and GPA was significant (b = −7.1; 95%CI − 12.5 to − 1.8) (Table 4). In addition, the interaction term for consistency of interest and GPA was significant (b = 4.9; 95%CI − 1.0 to 10.9) (Table 5). These interactions of the subscales remained significant after adjusting for the covariates. To visualize the effect modification by GPA more clearly, we divided GPA into categories (upper, middle, and lower), corresponding to the three tertiles, and showed the association restricted to each in Figures 2 and 3. That is, as shown in Figure 2, the association between perseverance of effort and depressive symptoms was significantly stronger among the students with higher academic achievements. On the other hand, as shown in Figure 3, the association between consistency of interest and depressive symptoms was significantly stronger among the students with lower academic achievements.

**Table 3.** Interaction between the total grit score and academic performance on depressive symptoms.

	Crude		Adjusted^a^	
Variable	b (95%CI)	p value	b (95%CI)	p value
Total grit score	−4.2 (−6.3, −2.1)	<0.001	−4.4 (−6.5, −2.3)	<0.001
GPA	−4.0 (−9.5, 1.5)	0.151	−3.2 (−8.9, 2.5)	0.274
Total grit score × GPA	0.69 (−8.4, 9.8)	0.882	1.7 (−7.4, 10.8)	0.718

Abbreviations: CI = confidence interval; GPA = Grade Point Average. ^a^Adjusted for sex, age and survey year.

**Table 4.** Interaction effect between perseverance of effort and academic performance on depressive symptoms.

	Crude		Adjusted^a^	
Variable	b (95%CI)	p value	b (95%CI)	p value
Perseverance of effort	−3.7 (−5.3, −2.0)	<0.001	−3.7 (−5.4, −2.1)	<0.001
GPA	−3.9 (−9.3, 1.5)	0.157	−3.2 (−8.8, 2.5)	0.272
Perseverance of effort × GPA	**−7.1 (−12.5, −1.8)**	**0.009**	**−7.1 (−12.4, −1.7)**	**0.010**

Abbreviations: CI = confidence interval; GPA = Grade Point Average. ^a^Adjusted for sex, age and survey year.

**Table 5.** Interaction effect between consistency of interest and academic performance on depressive symptoms.

	Crude		Adjusted^a^	
Variable	b (95%CI)	p value	b (95%CI)	p value
Consistency of interest	−1.5 (−3.1, 0.1)	0.066	−1.7 (−3.3, −0.1)	0.042
GPA	−8.4 (−14.1, −2.6)	0.005	−7.4 (−13.4, −1.4)	0.016
Consistency of interest × GPA	4.9 (−1.0, 10.9)	0.107	5.5 (−0.4, 11.4)	0.071

Abbreviations: CI = confidence interval; GPA = Grade Point Average. ^a^Adjusted for sex, age and survey year.

## Discussion

This study found that, among medical students, the total grit score and the two subscales were all significantly inversely associated with depressive symptoms. Furthermore, we revealed that academic performance moderated the association between perseverance of effort and depressive symptoms and the association between consistency of interest and depressive symptoms and that these moderating effects were inverse.

A previous study reported the negative association between the total grit score and depression among medical students [31]; however, it did not show the results regarding the subscales. A study by Zhang et al. suggested that both perseverance of effort and consistency of interest were negatively associated with depression among university students [35]. In addition, Akaishi et al. found that among 221 medical residents the total grit score and perseverance of effort were both negatively associated with depressive symptoms (odds ratio (OR) for the total grit score = 0.39, 95%CI 0.20 to 0.77; OR for perseverance of effort = 0.42, 95%CI 0.23 to 0.75) and that the consistency of interest appeared to be associated with depressive symptoms to some extent although the result was not statistically significant (OR = 0.61, 95%CI 0.36 to 1.05) [27]. In the present study, both perseverance of effort and consistency of interest were found to be inversely associated with depressive symptoms and the association between perseverance of effort and depression appeared to be greater (coefficient (b) = −3.7; 95%CI − 5.3 to − 2.1) than that between consistency of interest and depression (b = −1.8, 95%CI − 3.5 to − 0.2). Consistency of interest, which involves constantly demonstrating interest [26], might contribute to maintaining students’ goals even under stressful circumstances and act as a protective factor against depression. Moreover, perseverance of effort, which entails showing a high degree of persistence even after experiencing setbacks or failures [26], might alleviate psychosocial stressors inducing depressive symptoms.

This study indicated that the association between perseverance of effort and depressive symptoms was stronger among students with higher academic achievements while the association between consistency of interest and depressive symptoms was stronger among students with lower academic achievements. In other words, perseverance of effort appeared to be more essential for reducing depressive symptoms of students with higher academic performance, and consistency of interest seemed to be more important for decreasing depressive symptoms of students with lower academic performance. Interestingly, the inverse moderating effects by the two subscales may have cancelled each other out and led to no significant moderating effect by the total grit score. A systematic review of the studies that focused on the relationship between grit and academic performance, in which the study participants were students attending several levels of education (primary school to university) and more than half of them were college or university students, reported that the association between perseverance of effort and academic performance was stronger than that between consistency of interest and academic performance [42]. Therefore, for students with higher or middle academic performance, perseverance of effort may encourage them to study eagerly, promote their academic success, and alleviate depression-inducing psychosocial stressors, including academic burden.

A study focusing on the students on academic probation in a university reported that consistency of interest was significantly associated with academic improvement whereas perseverance of effort showed no significant association with it [57]. In addition, another study suggested that the students with high consistency of interest were capable of maintaining their initial enthusiasm for their goals even in the face of difficulties [51]. Thus, for students with particularly low academic performance, consistency of interest may contribute both to maintaining their initial motivation for becoming medical doctors and to diminishing risk factors for depression, including stressful academic difficulties.

Several limitations of the current study must be acknowledged. First, this study focused on only second-year students, previous studies having suggested that depression prevalence was especially high in earlier years in medical school and that depressive symptoms had worsened from first year to second year [1,10,11]. Students experience various changes in lifestyle and learning environment in the earlier years after entering university. In later years, they will encounter other difficulties related with highly professional subjects in medical degree courses. Therefore, studies involving students in other years are also important. Second, since this study was conducted at just one university, the findings of it may not be transferable to medical students in other universities. Because TMDU is located in the capital of Japan, the environmental influences on students may be different from those in universities in rural areas. Thus, multicenter studies are warranted to confirm the findings. Third, there may be sampling bias in this study in which 177 out of 434 students participated. Some of the participants might have participated in the study in order to try to draw attention to their own mental distress; and some of the non-participants might have avoided participation for fear of being perceived as mentally vulnerable. Fourth, the CES-D scores are not the same as clinically diagnosed depression. Fifth, the results of the present study may have been influenced by the coronavirus disease 2019 (COVID-19) pandemic. However, in spite of the influence of the COVID-19 pandemic, likely changing over time, the results of the model, adjusted for covariates, including survey year, were similar to those of the crude models. Finally, this was a cross-sectional study and was not able to elucidate causal relationships. Longitudinal studies are needed to clarify whether and how grit ameliorates depressive symptoms over time.

Our findings have the following implication. Previous studies have not clarified whether and how students’ grit changes through the course of their medical education [43,58,59]. However, grit appears to be a cultivable psychological factor [60–63]. Although evidence is lacking to date that interventions to enhance grit are able to alleviate depressive symptoms, nurturing grit may be a noteworthy way to reduce the risk of depression. Our findings found that the especially essential factors for alleviating depressive symptoms might be perseverance of effort among students with higher or middle academic achievements and consistency of interest among students with lower academic outcomes. Therefore, tailoring the fostering of different components of grit, especially by academic performance, may be a more effective way to prevent depression among medical students.

## Conclusions

This study reveals a novel aspect of the association between grit and depressive symptoms from the viewpoint of academic performance among medical students. These findings will contribute to future research on the mental health of medical students.
